# The effect of moderate weight loss, with or without (1, 3)(1, 6)-β-glucan addition, on subcutaneous adipose tissue inflammatory gene expression in young subjects with uncomplicated obesity

**DOI:** 10.1007/s12020-018-1619-z

**Published:** 2018-05-08

**Authors:** Marek Strączkowski, Agnieszka Nikołajuk, Radosław Majewski, Remigiusz Filarski, Magdalena Stefanowicz, Natalia Matulewicz, Monika Karczewska-Kupczewska

**Affiliations:** 1 Department of Prophylaxis of Metabolic Diseases, Institute of Animal Reproduction and Food Research, Polish Academy of Sciences, Olsztyn, Poland; 2 Department of Metabolic Diseases, Medical University of Bialystok, Bialystok, Poland

**Keywords:** Obesity, Inflammation, Insulin sensitivity, Low-calorie diet, (1, 3)(1, 6)-β-glucan

## Abstract

**Purpose:**

Obesity is characterized by insulin resistance and low-grade systemic and adipose tissue (AT) inflammation. It remains unclear whether beneficial effects of weight loss are related to AT inflammation. We aimed to assess the effect of weight loss during low-calorie diet on insulin sensitivity, AT expression of genes associated with inflammation in young subjects with obesity. Furthermore, we estimated the effects of immunomodulatory (1, 3)(1, 6)-β-glucan (BG) on the above parameters.

**Methods:**

The study group comprised 52 subjects with obesity. Twelve-week dietary intervention was applied, with randomization to receive or not 500 mg BG daily. Euglycemic hyperinsulinemic clamp, subcutaneous AT biopsy were performed before and after the program. Twenty normal-weight subjects, examined at baseline, served as a control group.

**Results:**

At baseline, obese subjects had lower insulin sensitivity, lower AT *ADIPOQ*, *JAK1*, and *JAK2* expression and higher AT expression of *LEP*, *IL6ST*, *STAT3*, *MIF*, *CCL2*, *MMP9*, and *IL18*. Forty obese subjects completed dietary intervention program, which resulted in 11.3% weight loss and 27% increase in insulin sensitivity (both *p* < 0.0001). AT *IL6R*, *IL6ST*, *JAK1*, and *JAK2* expression increased, whereas *MIF*, *CCL2*, *MMP9*, and *IL18* gene expression did not change in response to weight loss. BG addition had no effect on any of the parameters studied.

**Conclusions:**

Our data indicate that reduction in AT inflammation is not required for an improvement in insulin action during weight loss in subjects with uncomplicated obesity. BG does not have effects during dietary intervention.

## Introduction

Obesity is associated with an increased risk of type 2 diabetes, dyslipidemia, hypertension, cardiovascular disease, different forms of cancer [1]. Insulin resistance, i.e. decreased biological response to insulin, and low-grade systemic and adipose tissue (AT) inflammation play a role in the development of metabolic complications associated with the excess of body fat [2, 3]. In obesity, increased circulating proinflammatory cytokine concentrations are observed [3]. Inflammatory factors may directly inhibit insulin signaling and thus induce insulin resistance [4]. AT contributes to systemic inflammation in obesity through synthesis and secretion of multiple proinflammatory molecules [5]. Adipocytes from both visceral and subcutaneous AT display proinflammatory profile in obesity [6]. However, AT is infiltrated by macrophages and other immune cells, which also promote local and systemic inflammation [7]. Macrophage migration inhibitory factor (MIF), monocyte chemoattractant protein 1 (MCP-1), matrix metalloproteinase 9 (MMP-9), and interleukin 18 (IL-18) are important factors regulating macrophage functions [7, 8].

Glycoprotein 130 (gp130) cytokines regulate inflammatory processes. This family comprises a group of proteins, which include interleukin 6 (IL-6) among others, that induce their signal through association with cell-surface transmembrane receptor (like IL-6R) and signal transduction membrane-bound gp130, also known as IL-6 signal transducer (IL-6ST) [9], common for all cytokines. Intracellular gp130 signaling involves Janus Kinase/Signal Transducer and Activator of Transcription (JAK/STAT) pathway. Phosphorylated STAT molecules translocate into the nucleus and activate gene transcription. The main negative feedback regulator of the gp130 signaling is Suppressor of Cytokine Signaling 3 (SOCS3), which is a STAT3 target gene and is transcriptionally upregulated after cellular gp130 activation by IL-6 [10]. Both IL-6R and gp130 are present in the blood in the soluble forms (sIL-6R and sgp130, respectively). It was reported that sgp130 may inhibit intracellular gp130 signaling [11]. In our previous studies we demonstrated that sgp130 was inversely related to insulin action, which may suggest an inhibition of intracellular gp130 signaling in insulin-resistant conditions [12]. However, little is known about the expression of gp130 signaling genes in metabolic tissues in humans, especially in AT.

Weight loss is an effective method to combat the detrimental effects of obesity. For instance, massive weight loss after bariatric surgery resulted in type 2 diabetes remission in most of the patients [13]. Moderate weight loss of 5–10% is also associated with health benefits. Weight loss improves insulin sensitivity [14, 15]. However, its effects on AT inflammation are less certain, as conflicting data have been reported [16–20]. Thus, it remains unclear to what extent improvement in insulin sensitivity during weight loss is due to the reduction in AT inflammation. Furthermore, the effect of weight loss on AT gp130 signaling gene expression has not been extensively studied so far.

Dietary fiber consumption is inversely related to type 2 diabetes risk [21, 22]. However, it is a complex substance and it is unclear which ingredient is responsible for this beneficial metabolic effect. β-glucans (BGs) represent biologically active sort of dietary fiber. BGs are polysaccharides consisting of D-glucose monomers linked by β-glycosidic bonds [23]. They are found at a high level in the cell wall of fungi, yeast, oat, barley, as well as bacteria. Their structures are diverse with fungal and yeast BGs being β-1,3 linkage branched by 1,6 ((1, 3) (1,6) BG), while oat and barley BGs linked by linear 1,3 and 1,4 bonds ((1,3) (1, 4) BG) [24]. Yeast-derived (1, 3) (1, 6) BG has immunomodulatory properties [23]. BGs also improve metabolic disturbances associated with obesity [25]. Thus, one may hypothesize that it may influence systemic and AT inflammation during weight loss.

The aim of the present study was to assess the effect of moderate weight loss during low-calorie diet on insulin sensitivity, AT and peripheral blood mononuclear cells (PBMC) inflammatory gene expression in young subjects with obesity without concomitant diseases. Furthermore, we estimated the effects of (1, 3)(1, 6) BG on the above parameters.

## Materials and methods

### Participants

The study group comprised 52 subjects with marked overweight or obesity (BMI > 28 kg/m^2^, 27 males and 25 females). Twenty normal-weight subjects (8 males and 12 females) served as a control group. All study participants were nonsmokers, without impaired glucose tolerance/diabetes, hypertension, cardiovascular disease, dyslipidemia, thyroid or other hormonal dysfunction, liver or renal failure, neoplasms or other serious disease, morbid obesity, and were not taking drugs known to affect carbohydrate and lipid metabolism, body weight and also hormonal and any other drugs. Body of the subjects had remained stable (±1 kg) for at least three months prior to the study. Participants underwent clinical examination and appropriate laboratory tests. Subjects were excluded if they had any inflammatory disease within the last three months. All subjects had no clinical and laboratory signs of inflammation and had not taken anti-inflammatory drugs within the last three months. A standard oral glucose tolerance test (OGTT) was performed and all subjects had normal glucose tolerance according to World Health Organization criteria. All the studies were performed after overnight fast.

The study protocol was approved by the local ethics committee of the Medical University of Białystok, Poland. A written informed consent was obtained from all individual participants included in the study. The study has been registered at www.clinicaltrials.gov (NCT01393210).

### Study protocol

Subjects with overweight/obesity underwent a 12-week dietary intervention program, which consisted of individually planned low-calorie diet (20 kcal per kg of proper body weight). The ideal body weight was calculated for the Broca formula. The sources of energy in the diet were carbohydrate 55–60%, fat 25%, and protein 15–20%. Additionally, overweight/obese subjects were randomly assigned to receive or not (1, 3)(1, 6) BG preparation (BETA-GLUKAN 1.3-1.6 D, 500 mg, Laboratoria Natury, Lublin, Poland) once daily as an addition to low-calorie diet. Each study arm (i.e., no BG and BG) comprised 26 subjects (no BG, 13 males and 13 females; BG, 14 males and 12 females). Participants received a detailed instruction abut low-calorie diet. The body weight changes were assessed every two weeks. The compliance was also assessed every two weeks by qualified dieticians—each participant reported non-adherence to the prescribed diet in a diary. This BG preparation is used as a non-prescription diet supplement. All analyses described below were performed before and after dietary intervention. Normal-weight subjects were examined only at baseline.

### Anthropometric measurements

Body mass index (BMI) was calculated as body weight × height^−2^ and expressed in kilograms per square meter. The waist circumference was measured at the smallest circumference between the rib cage and iliac crest, with the subject in the standing position. The percentage of body fat was calculated by bioelectric impedance analysis using the Tanita TBF-511 Body Fat Analyzer (Tanita, Tokyo, Japan).

### Insulin sensitivity

Insulin sensitivity was measured with 2 h euglycemic hyperinsulinemic clamp technique, as described previously [26]. The rate of whole-body glucose uptake (M value) was calculated as the mean glucose infusion rate during the last 40 min of the clamp, corrected for the glucose space, and divided by fat-free mass (ffm).

### AT biopsy

Subcutaneous AT biopsy was obtained from the umbilical region using biopsy needle and collected to 1 ml of RNA stabilization reagent (Allprotect Tissue Reagent, Qiagen GmbH, Hilden, Germany), as described [27–29]. Tissues were kept at −80 °C until analyses.

### PBMC isolation

PBMC were isolated from fresh blood using 10 mL BD Vacutainer® CPT™ Cell Preparation Tube (Becton Dickinson AG, NJ, USA) with sodium citrate, following the instructions of the manufacturer. All procedures were carried out within two hours from blood collection. Pellets were frozen and stored at −80 °C prior to RNA isolation [28].

### Biochemical analyses

Plasma glucose was measured immediately by the enzymatic method using a glucose analyzer (YSI 2300 STAT PLUS). Serum insulin was measured with the monoclonal immunoradiometric assay (IRMA; DIAsource ImmunoAssays S.A., Nivelles, Belgium) with the sensitivity of 1 μIU/mL and with intra-assay and interassay coefficients of variation below 2.2 and 6.5%, respectively. Serum free fatty acids (FFA) were assayed using a commercially available kit (Wako Chemicals, Richmond, VA). Serum total cholesterol, triglycerides (TG), HDL-cholesterol and LDL-cholesterol were assessed by the colorimetric assays using the autoanalyzer Cobas c111 (Roche Diagnostics, Mannheim, Germany). Serum high-sensitive C-reactive protein (hsCRP) was measured with particle enhanced immunonephelometry (Dade Behring, Marburg, Germany).

Serum adiponectin and leptin were measured with RIA kits (Millipore, Inc. St. Charles, MO, USA). Serum high-sensitive IL-6, sIL-6R, sgp130, MIF, MCP-1, and MMP-9 concentrations were measured with ELISA kits (R&D Systems Inc, Minneapolis, MN, USA). Serum IL-18 was measured with an ELISA kit (MBL Co., Ltd., Nagoya, Aichi, Japan).

### Isolation of mRNA from AT and PBMC and determination of gene expression

Total RNA was isolated from AT and PBMC as described [27–29]. RNA was treated with Turbo DNA-free Kit (Ambion, Austin, TX) to remove any trace of DNA. Assessment of RNA quantity and quality were confirmed using an Agilent Technologies 2100 Bioanalyzer and RNA 6000 Nano LabChip kit (Agilent, Mountain View, CA). RNA purity (A260/A280 ratio) was assessed by using a NanoDrop spectrophotometer (NanoDrop 2000, Thermo Scientific Inc., Wilmington, DE). Reverse transcription was performed using 200 ng of total RNA to synthesize the first strand of cDNA using the QuantiTect Reverse Transcription kit (Qiagen, Austin, TX) [27–29].

AT mRNA expression of ADIPOQ, *LEP* and genes of IL-6 signaling and inflammatory factors were analyzed with quantitative Real-Time PCR. In PBMC we analyzed mRNA expression of IL-6 signaling and inflammatory factors. The samples were quantified with the Light Cycler 480 II Real-Time PCR Instrument (Roche Diagnostics, GmbH, Mannheim, Germany) using gene specific primers and probes (Table 1). All samples were run in triplicate and average values were calculated. All results were normalized to the levels of the phosphoglycerate kinase 1 (*PGK1*), as its expression was the most stable in our study group from the few possible house-keeping genes tested, and relative quantification was calculated using the ΔΔCt formula cycle threshold (Ct).

**Table 1:** Assay information

Gene symbol	Forward primer sequence	Reverse primer sequence
*ADIPOQ*	GGTGAGAAGGGTGAGAAAGGA	TTTCACCGATGTCTCCCTTAG
*LEP*	GCCTTGAAGGTCACTCTTCCT	CATGCAATGCTCTTCAATCC
*IL6*	CCAGAGCTGTGCAGATGAGT	GGGTCAGGGGTGGTTATTG
*IL6R*	GATTCTGCAAATGCGACAAG	TGTGGGCAGTGGTACTGAAG
*IL6ST*	TGTTGGCAAATCAGATGCAG	AAGATCCATTACAGGGTGAGTAGC
*JAK1*	AATGGCTGTCATGGTCCAAT	TACATCCCCTCCTCGCTTC
*JAK2*	CAGGAACAAGATGTGAACTGTTTC	CCCATGCAGAGTCTTTTTCAG
*STAT3*	TCCTGAAGCTGACCCAGGTA	GGTCGTTGGTGTCACACAGAT
*SOCS3*	GACTTCGATTCGGGACCAG	AACTTGCTGTGGGTGACCAT
*MIF*	GAAGTCAGGCACGTAGCTCAG	GGCAGAAGGACCAGGAGAC
*CCL2*	AGTCTCTGCCGCCCTTCT	GTGACTGGGGCATTGATTG
*MMP9*	ATCCGGCACCTCTATGGTC	CAGACCGTCGGGGGAG
*IL18*	CAACAAACTATTTGTCGCAGGA	TGCCACAAAGTTGATGCAAT
*NFKB1*	ACCCTGACCTTGCCTATTTG	AGCTCTTTTTCCCGATCTCC
*NFKB2*	CCCATCCATGACAGCAAAT	CTTGTCACAAAGCAGATAAACTTCA
*MAPK8*	GGGAACACACAATAGAAGAGTGG	TGCCCCCGTATAACTCCAT
*PGK1*	GGAGAACCTCCGCTTTCAT	GCTGGCTCGGCTTTAACC

### Statistical analysis

The statistics were performed with the STATISTICA 12.5 (Statsoft, Krakow, Poland). All data are presented as mean ± SD. The variables, which did not have normal distribution were log-transformed before analyses. For the purpose of the data presentation, absolute values are shown in the Results. Differences between the groups were analyzed with the unpaired Student’s *t*-test for continuous variables and with Chi-square test for categorical variables. Differences in estimated parameters before and after weight loss program were assessed with the paired Student’s *t*-test. Relationships between variables were studied with the Pearson product moment correlation analysis and with multiple regression analysis. The level of significance was accepted at *p* value lower than 0.05.

## Results

### Baseline differences between normal-weight and obese subjects

By definition, normal-weight and obese subjects differed in anthropometric parameters (all *p* < 0.05). Obese subjects had also higher systolic and diastolic blood pressure, fasting glucose, insulin, FFA, total and LDL-cholesterol, TG, hsCRP, serum leptin, IL-6, sgp130, IL-18 and lower insulin sensitivity, HDL-cholesterol, and serum adiponectin in comparison to normal-weight subjects (all *p* < 0.05; Table 2).

**Table 2:** Baseline characteristics of the study groups

	Normal weight (*n* = 20)	Overweight/obese (*n* = 52)	*p* value
	Basal clinical and laboratory parameters		
Age	23.50 ± 1.79	32.04 ± 7.88	0.0009
Body weight (kg)	68.08 ± 9.21	98.81 ± 14.82	<0.000001
BMI (kg/m^2^)	22.38 ± 2.34	32.57 ± 2.99	<0.000001
Waist circumference (cm)	80.75 ± 5.72	107.08 ± 9.05	<0.000001
% body fat	23.73 ± 8.01	38.31 ± 5.87	<0.000001
Systolic BP (mmHg)	120.50 ± 8.25	132.12 ± 10.59	0.00004
Diastolic BP (mmHg)	73.85 ± 5.82	82.28 ± 8.17	0.00008
Fasting plasma glucose (mg/dL)	81.61 ± 6.45	88.18 ± 5.43	0.0001
Fasting serum insulin (μIU/mL)	10.57 ± 6.02	14.99 ± 4.97	0.0037
M (mg/kg ffm/min)	9.77 ± 3.36	6.59 ± 2.97	0.0004
Fasting serum FFA (mmol/L)	0.52 ± 0.18	0.68 ± 0.20	0.031
Cholesterol (mg/dL)	169.60 ± 31.85	193.90 ± 29.81	0.0033
TG (mg/dL)	74.20 ± 32.66	116.44 ± 58.57	0.0034
HDL-cholesterol (mg/dL)	66.83 ± 14.59	53.06 ± 12.93	0.00038
LDL-cholesterol (mg/dL)	92.62 ± 25.23	122.07 ± 31.73	0.0007
hsCRP (mg/L)	0.36 ± 0.25	1.28 ± 0.77	0.000003
Serum adipokine and cytokine concentrations			
Adiponectin (μg/mL)	14.29 ± 6.61	11.27 ± 4.74	0.047
Leptin (ng/mL)	5.93 ± 3.54	19.75 ± 9.51	<0.000001
hsIL-6 (pg/mL)	1.09 ± 0.51	1.64 ± 0.59	0.00007
sIL-6R (ng/mL)	60.38 ± 19.83	56.83 ± 15.11	0.44
sgp130 (ng/mL)	319.10 ± 43.73	356.95 ± 70.82	0.036
MIF (ng/mL)	32.88 ± 7.59	36.52 ± 10.58	0.20
MCP-1 (pg/mL)	341.30 ± 69.58	372.51 ± 71.28	0.13
MMP-9 (ng/mL)	713.34 ± 245.51	802.71 ± 422.60	0.39
IL-18 (pg/mL)	203.00 ± 47.95	283.22 ± 77.06	0.00026
AT gene expression (A.U.)			
*ADIPOQ*	1.67 ± 0.67	1.00 ± 0.48	0.00008
*LEP*	0.70 ± 0.23	1.02 ± 0.60	0.033
*IL6*	0.93 ± 0.77	0.89 ± 0.57	0.86
*IL6R*	0.96 ± 0.54	1.00 ± 0.39	0.77
*IL6ST*	0.62 ± 0.31	1.00 ± 0.34	0.0001
*JAK1*	1.41 ± 0.67	1.00 ± 0.28	0.0005
*JAK2*	1.55 ± 1.03	1.04 ± 0.41	0.0034
*STAT3*	0.52 ± 0.10	1.02 ± 0.27	<0.000001
*SOCS3*	1.13 ± 0.60	0.99 ± 0.78	0.46
*MIF*	0.36 ± 0.11	0.96 ± 0.24	<0.000001
*CCL2*	0.39 ± 0.19	0.94 ± 0.50	0.000007
*MMP9*	0.21 ± 0.23	0.97 ± 0.87	0.0005
*IL18*	0.38 ± 0.20	1.00 ± 0.63	0.00005

*A.U* arbitrary units, *BP* blood pressure

AT expression of *ADIPOQ JAK1* and *JAK2* was lower, whereas expression of *LEP*, *IL6ST*, *STAT3*, *MIF*, *CCL2* (encoding MCP-1), *MMP9*, and *IL18* was higher in the obese in comparison to the normal-weight group (Table 2). PBMC expression of IL-6 signaling genes and inflammatory factors did not differ between the groups (data not shown).

### Effect of dietary intervention on body weight and composition, insulin sensitivity, blood pressure, and laboratory parameters

A total of 40 out of 52 subjects completed the dietary intervention program, 22 in no BG and 18 in BG group. The drop outs were due to the non-adherence to the prescribed diets. There was no significant interaction between the study group and program completion (Chi-square *p* = 0.32). No baseline differences in any of the parameters studied between no BG and BG groups were observed. No relevant adverse effects was observed during the study.

Dietary intervention resulted in a significant weight loss of ~11.3% (*p* < 0.0001). This decrease was mainly due to a decrease in fat mass (~−23.5%, *p* < 0.0001), although fat-free mass also decreased slightly (~−3.6%, *p* < 0.0001). It was accompanied by a decrease in waist circumference, systolic and diastolic blood pressure, fasting serum insulin, cholesterol, TG, and hsCRP (all *p* < 0.05; Table 3). All these effects were similar whether or not subjects received BG (all *p* < 0.05 in no BG and BG subgroups). Fasting plasma glucose decreased significantly when the entire group was analyzed together (*p* = 0.008); however, this decrease did not reach statistical significance when no BG (*p* = 0.057) and BG groups (*p* = 0.068) were analyzed separately (Table 3). Insulin sensitivity increased by ~27% from the baseline value, this increase was comparable in both groups (*p* = 0.001; Table 3).

**Table 3:** Effect of dietary intervention on body weight and composition, insulin sensitivity, blood pressure, and laboratory parameters

	No BG (*n* = 22)				BG (*n* = 18)			
	Before	After	Δ	*p*	Before	After	Δ	*p*
Body weight (kg)	100.97 ± 15.70	89.79 ± 14.25	−11.18	<0.000001	96.61 ± 12.73	85.44 ± 11.19	−11.17	<0.000001
BMI (kg/m^2^)	33.50 ± 3.06	29.83 ± 2.85	−3.67	<0.000001	32.19 ± 2.95	28.48 ± 2.82	−3.71	<0.000001
Waist (cm)	107.32 ± 11.51	98.59 ± 10.27	−8.73	<0.000001	106.94 ± 6.10	96.83 ± 5.70	−10.11	<0.000001
Fat mass (kg)	40.75 ± 8.54	31.92 ± 7.39	−8.83	<0.000001	36.42 ± 6.46	26.98 ± 7.06	−9.44	<0.000001
Fat-free mass (kg)	61.22 ± 10.96	58.61 ± 11.32	−2.61	0.000005	60.20 ± 11.45	58.49 ± 12.11	−1.71	0.001
Systolic BP (mmHg)	131.71 ± 9.20	124.67 ± 9.87	−7.04	0.004	131.69 ± 12.06	124.12 ± 10.51	−7.57	0.014
Diastolic BP (mmHg)	82.05 ± 8.77	79.05 ± 8.84	−3.00	0.046	83.35 ± 8.59	77.00 ± 7.90	−6.35	0.02
Fasting plasma glucose (mg/dL)	88.77 ± 5.71	86.55 ± 6.78	−2.22	0.057	87.32 ± 5.27	83.48 ± 7.92	−3.84	0.068
Fasting serum insulin (μIU/mL)	15.28 ± 5.04	11.55 ± 2.65	−3.73	0.0004	15.01 ± 6.46	10.02 ± 3.27	−4.99	0.003
M (mg/kg ffm/min)	6.53 ± 2.92	8.59 ± 3.54	2.06	0.021	6.38 ± 3.17	7.77 ± 2.77	1.39	0.007
Fasting serum FFA (mmol/L)	0.72 ± 0.21	0.85 ± 0.30	0.13	0.22	0.64 ±0.17	0.71 ± 0.35	0.07	0.52
Cholesterol (mg/dL)	190.91 ± 25.30	175.36 ± 28.57	−15.55	0.005	185.82 ± 28.19	160.18 ± 32.61	−25.64	0.0006
TG (mg/dL)	107.20 ± 56.69	77.20 ± 37.08	−30.00	0.007	113.12 ± 52.67	81.12 ± 20.25	−32.00	0.009
HDL-cholesterol (mg/dL)	49.50 ± 7.21	49.17 ± 8.13	−0.33	0.87	53.62 ± 12.27	51.31 ± 11.40	−2.31	0.27
LDL-cholesterol (mg/dL)	122.07 ± 28.09	107.99 ± 35.92	−14.08	0.14	115.76 ± 30.79	103.29 ± 32.52	−12.47	0.25
hs CRP (mg/L)	1.12 ± 0.89	0.71 ± 0.51	−0.41	0.008	1.32 ± 0.77	0.79 ± 0.63	−0.53	0.003

Δ represents difference after vs before dietary intervention within the No BG and BG groups

*p* values refer to the differences before and after dietary intervention within the No BG and BG groups

### Effect of dietary intervention on serum adipokine and cytokine concentrations

Serum leptin, MCP-1, and IL-18 concentrations decreased significantly after dietary intervention in a similar degree in both groups (all *p* < 0.05; Table 4). We observed also a decrease in serum IL-6 when the entire group was analyzed together (*p* = 0.048); however, it was not significant in no BG and BG groups assessed separately (Table 3). Serum adiponectin, sIL-6R, sgp130, MIF, and MMP-9 did not markedly change after weight loss (Table 4).

**Table 4:** Effect of dietary intervention on serum adipokine and cytokine concentrations

	No BG (*n* = 22)				BG (*n* = 18)			
	Before	After	Δ	*p*	Before	After	Δ	*p*
Adiponectin (μg/mL)	10.49 ± 4.42	12.36 ± 5.51	1.87	0.15	12.20 ± 5.22	11.12 ± 4.58	−1.08	0.32
Leptin (ng/mL)	20.01 ± 8.40	10.16 ± 6.09	−9.85	0.000001	18.59 ± 10.67	7.15 ± 5.43	−11.44	0.000002
hs IL-6 (pg/mL)	1.69 ± 0.71	1.41 ± 0.37	−0.28	0.17	1.57 ± 0.44	1.47 ± 0.45	−0.10	0.20
sIL-6R (ng/mL)	57.20 ± 13.64	55.55 ± 12.49	−1.65	0.50	56.12 ± 17.64	54.75 ± 17.41	−1.37	0.40
sgp130 (ng/mL)	362.57 ± 74.09	347.44 ± 60.35	−15.13	0.28	350.18 ± 71.18	342.18 ± 59.31	−8.00	0.40
MIF (ng/mL)	35.87 ± 10.61	34.06 ± 15.65	−1.81	0.57	37.82 ± 11.40	39.19 ± 16.45	1.37	0.80
MCP-1 (pg/mL)	374.67 ± 64.86	341.93 ± 58.18	−32.74	0.0006	359.57 ± 67.22	325.16 ± 71.15	−34.41	0.0002
MMP-9 (ng/mL)	814.18 ± 428.91	643.52 ± 319.81	−170.66	0.14	810.99 ± 429.82	690.18 ± 203.58	−120.81	0.15
IL-18 (pg/mL)	291.63 ± 85.81	259.71 ± 54.38	−31.92	0.023	273.23 ± 66.56	231.43 ± 52.87	−41.80	0.004

Δ represents difference after vs before dietary intervention within the No BG and BG groups

*p* values refer to the differences before and after dietary intervention within the No BG and BG groups

### Effect of dietary intervention on AT and PBMC gene expression

**Fig. 1:**
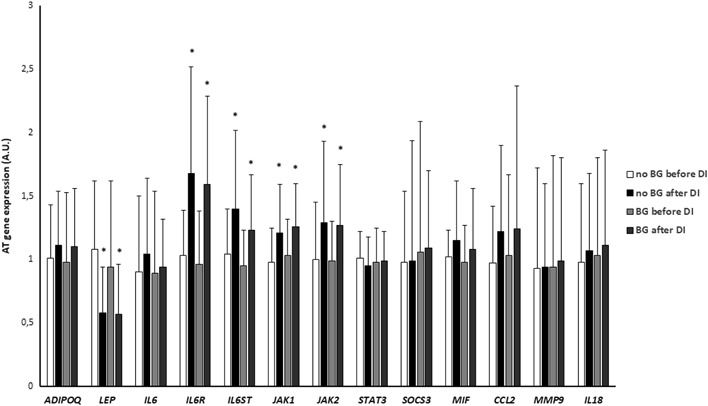
Effect of dietary intervention on AT gene expression in no BG (*n* = 22) and BG (*n* = 18) groups. A.U., arbitrary units; DI, dietary intervention. *p < 0.05 vs before dietary intervention

Dietary intervention resulted in a significant decrease in AT *LEP* expression (*p* < 0.0001) and an increase in AT *IL6R*, *IL6ST*, *JAK1*, and *JAK2* expression (all *p* < 0.05). All these effects were similar in both groups (Fig. 1). AT expression of *ADIPOQ*, *IL6*, *STAT3*, *SOCS3*, *MIF*, *CCL2*, *MMP9*, and *IL18* did not change during dietary intervention (Fig. 1).

PBMC gene expression did not change after weight loss both in no BG and BG groups (data not shown).

### Correlations between the analyzed variables

Serum hsCRP and leptin were related to BMI both before (*r* = 0.55 and *r* = 0.67, respectively, both *p* < 0.0001) and after dietary intervention (both *r* = 0.46, both *p* = 0.003). Serum adiponectin was related to insulin sensitivity before dietary intervention (*r* = 0.33, *p* = 0.011). As in serum, AT *LEP* was related to BMI both before and after dietary intervention (*r* = 0.30, *p* = 0.02 and *r* = 0.39, *p* = 0.013, respectively). AT *STAT3*, *MIF*, *CCL2*, *MMP9*, and *IL18* were positively associated, whereas AT *JAK1* and *JAK2* were negatively associated with BMI in the baseline state (before dietary intervention; all *p* < 0.05). AT *STAT3*, *SOCS3*, *MIF*, *CCL2*, *MMP9*, and *IL18* were inversely related to insulin sensitivity before dietary intervention (all *p* < 0.05). In multiple regression analysis, only correlations of *STAT3* and *SOCS3* with insulin sensitivity before dietary intervention remained significant after adjustment for BMI (*β* = −0.31, *p* = 0.049 and *β* = −0.27, *p* = 0.038, respectively).

The changes in AT *JAK1* and *JAK2* expression during dietary intervention were positively related to the concurrent changes in *IL6R* and *IL6ST* (*JAK1*, *r* = 0.37, *p* = 0.019 and *r* = 0.44, *p* = 0.005, respectively; *JAK2*, *r* = 0.64, *p* < 0.0001 and *r* = 0.40, *p* = 0.011, respectively).

## Discussion

In the present study we observed the alterations in gp130 signaling and inflammatory gene expression in AT, but not in PBMC of obese subjects in comparison to normal-weight individuals. AT *STAT3* and *SOCS3* expression was independently and inversely related to insulin action. Dietary-induced moderate weight loss was associated with a significant increase in insulin sensitivity and associated clinical and metabolic parameters, as well as with the changes in AT expression of gp130 signaling genes. Addition of BG to low-calorie diet had no effect on any of the parameters studied.

Although the association between obesity and AT inflammation is well established [3, 5], the influence of the moderate weight loss on AT inflammatory parameters is less clear. There are studies indicating a reduction in AT inflammation after weight loss induced by bariatric surgery [16, 18] and energy-restricted diets [17]. In these bariatric surgery studies, over 20 kg weight loss was achieved and the patients were studied 3 to 24 months after treatment [16, 18]. In the study of Mraz et al very-low-calorie diet was applied to morbidly obese type 2 diabetic patients hospitalized for 2 weeks [17]. In contrast, no effect of weight loss on AT inflammation was also reported after Roux-en-Y gastric bypass, when type 2 diabetic patients were studied after 7% weight loss, which was achieved after mean of 13 days. However, a significant improvement in glycemic control was observed at the same time [19]. Similarly, in some studies using dietary intervention to induce weight loss, lack of changes in AT inflammation was observed [30]. In another study, 5% weight loss was associated with an increase in insulin sensitivity without changes in systemic or AT markers of inflammation [20]. Thus, it is likely that the effects of weight loss on AT inflammation are dependent on the initial degree of obesity, the presence of associated metabolic disorders, like type 2 diabetes, the degree and duration of weight loss. Subjects with morbid obesity are often studied, usually with concomitant type 2 diabetes. In contrast, we studied young subjects without morbid obesity and without disturbances of glucose tolerance. Our study subjects were also ~10 years younger and had ~5 kg/m^2^ lower mean BMI than the participants reported in the study of Magkos et al. [20]. Although we observed increased baseline AT expression of proinflammatory genes, they remained unaffected by diet-induced weight loss, even it was above 10% of the initial body weight. Our data indicate that relatively mild increases in AT inflammation occurring early in the course of obesity are not normalized by the moderate weight loss. Nevertheless, we observed a significant increase in insulin sensitivity of ~27%, together with improvements in blood pressure and lipid profile. Our data clearly indicate that the reduction in AT inflammation is not necessary for the improvement in insulin action occurring during weight loss and other factors likely contribute to this effect. It should be also noted that although initial AT expression of inflammatory genes was related to insulin sensitivity, all these correlations disappeared after adjustment for BMI. Similarly to many other studies [16–20], we examined subcutaneous AT only. However, it was demonstrated that inflammation persisted also in visceral AT after weight loss in mice [31].

PBMC gene expression were not different between normal-weight and obese groups at baseline and remained unaffected by weight loss. Baseline results are in agreement with our recent data obtained in a different study population [28]. Lack of effect of weight loss on PBMC expression of inflammatory genes was also reported by other researchers [18]. Together, it is unlikely that PBMC influence low-grade chronic inflammatory state in uncomplicated obesity.

Despite the aforementioned findings, we observed mild decrease in some, but not all, circulating inflammatory parameters. Serum hsCRP and IL-18 concentrations, although decreased, were not normalized by weight loss. Our data do not indicate AT or PBMC as the major contributors to the mild improvement in systemic inflammation related to weight loss. Neither baseline nor post-intervention serum concentrations of inflammatory proteins were related to the respective insulin sensitivity values. Additionally, the changes in systemic inflammation and insulin sensitivity during dietary intervention were not related to each other. These data further support the hypothesis about the lack of effect of inflammation on the improvement in insulin sensitivity during weight loss.

In contrast to other genes associated with inflammation, we observed an increase in AT *IL6ST* and *STAT3* a decrease in AT expression of *JAK1* and *JAK2* in the obese in comparison with the normal-weight group at baseline. Furthermore, AT *STAT3* and *SOCS3* were the only genes studied, which were independently associated with insulin sensitivity. In obesity, increased AT *IL6*, *IL6R* [32], and *SOCS3* mRNA expression [33] was observed, however, unchanged AT *IL6*, *IL6R* and *IL6ST* expression was also reported [18]. Besides inflammatory response, gp130 signaling regulates also lipolysis and fat oxidation [34]. It should also be noted that gp130-associated lipolysis was implicated in the development of insulin resistance [35].

Serum IL-6 concentrations decreased (nonsignificantly within no BG and BG subgroups), whereas serum sIL-6R and sgp130 remained unchanged after weight loss. The expression of *IL6R*, *IL6ST*, *JAK1*, and *JAK2* increased after weight loss. These findings indicate rather local AT role of gp130 signaling during weight loss. To our knowledge, no study has reported AT expression of all genes involved in gp130 signaling after weight loss so far. Both decreased [36] and unchanged [18] AT *IL6* expression after weight loss was demonstrated. Trachta et al observed decreased AT *IL6R* expression 12 and 24 months after bariatric surgery-induced massive weight loss. However, they also showed nonsignificant 26% increase in AT *IL6ST* at the same time [18]. As for other genes associated with inflammation, the observed differences may be due to the different subjects characteristics and the degree/duration of weight loss. Our data suggest that upregulation of gp130 signaling genes AT expression may be important for the weight loss-associated lipolysis in young subjects with uncomplicated obesity after moderate weight loss, however, they are not related to the concurrent improvement in insulin action. Our study does not identify the mechanism of the increase in insulin sensitivity during weight loss and further research is required to clarify this issue.

Gp130 cytokines share JAK/STAT signaling pathway with leptin. However, an increase in AT *JAK1* and *JAK2* expression was related only to the concurrent changes in *IL6R* and *IL6ST* and not to a decrease in AT *LEP* expression, which suggest rather a role for gp130 signaling in modulating these changes.

We also tested the possibility that the addition of BG to low-calorie diet may have beneficial metabolic and/or anti-inflammatory effects. Numerous BGs were used in human studies and were demonstrated some beneficial actions, however, there were differences in the effects on particular metabolic parameters and with different BGs. The beneficial of BGs on waist circumference, circulating inflammatory parameters [37], plasma lipids [38] and postprandial insulin response [39, 40] were observed. When Mediterranean hypocaloric diet with fiber intake 25–30 g/day was applied to obese individuals, a mild decrease in total cholesterol was observed after 1 month, although the decrease in body weight was less than 5% [41]. However, no effect of BGs on lipids was also reported [37] and the potential effect on blood pressure was restricted to subjects with the baseline BMI values above the median [39]. Furthermore, although oat BG decreases total and LDL-cholesterol in high doses, this effect is more pronounced in subjects with diabetes and/or high initial cholesterol [42]. Another study reported no effect of (1, 3) (1,6) BG on insulin sensitivity and the majority of inflammatory parameters during shorter treatment period (four weeks), without dietary intervention [43]. Also, no effect of oat BG (i.e., different than in the present study) as an addition to energy-restricted diet on weight loss, circulating lipids, and markers of appetite regulation was observed during three month treatment in overweight women [44]. We did not observed any effect of BG addition to low-calorie diet on weight loss, insulin sensitivity, systemic and AT inflammation, and other parameters. The dose used in our study was similar to the dose used for the most of the treatment period in another study, where some beneficial effects of BG were observed [37]. It is possible that during such profound metabolic changes occurring during weight loss, BG is not able to exert any additional beneficial effect and its potential immunomodulatory effect is not necessary in such conditions. Our data indicate that it is not justified to administer BG as an additional treatment during weight-reducing dietary intervention.

In conclusion, our data indicate that reduction in AT inflammation is not required for an improvement in insulin action during weight loss in subjects with uncomplicated obesity. BG does not have metabolic or anti-inflammatory effects during dietary intervention.
